# APOA5 deficiency causes hypertriglyceridemia by reducing amounts of lipoprotein lipase in capillaries

**DOI:** 10.1016/j.jlr.2024.100578

**Published:** 2024-06-15

**Authors:** Ye Yang, Robert J. Konrad, Michael Ploug, Stephen G. Young

**Affiliations:** 1 Department of Medicine, David Geffen School of Medicine, University of California, Los Angeles, CA, USA; 2 Department of Human Genetics, David Geffen School of Medicine, University of California, Los Angeles, CA, USA; 3 Lilly Research Laboratories, Eli Lilly and Company, Indianapolis, IN, USA; 4 Finsen Laboratory, Copenhagen University Hospital-Rigshospitalet, Copenhagen N, Denmark; 5 Finsen Laboratory, Biotech Research and Innovation Centre, University of Copenhagen, Copenhagen N, Denmark

**Keywords:** APOA5, lipoprotein lipase, hypertriglyceridemia, ANGPTL3/8, monoclonal antibody

## Abstract

Apolipoprotein AV (APOA5) deficiency causes hypertriglyceridemia in mice and humans. For years, the cause remained a mystery, but the mechanisms have now come into focus. Here, we review progress in defining APOA5’s function in plasma triglyceride metabolism. Biochemical studies revealed that APOA5 binds to the angiopoietin-like protein 3/8 complex (ANGPTL3/8) and suppresses its ability to inhibit the activity of lipoprotein lipase (LPL). Thus, APOA5 deficiency is accompanied by increased ANGPTL3/8 activity and lower levels of LPL activity. APOA5 deficiency also reduces amounts of LPL in capillaries of oxidative tissues (e.g., heart, brown adipose tissue). Cell culture experiments revealed the likely explanation: ANGPTL3/8 detaches LPL from its binding sites on the surface of cells, and that effect is blocked by APOA5. Both the low intracapillary LPL levels and the high plasma triglyceride levels in *Apoa5*^*−/−*^ mice are normalized by recombinant APOA5. Carboxyl-terminal sequences in APOA5 are crucial for its function; a mutant APOA5 lacking 40-carboxyl-terminal residues cannot bind to ANGPTL3/8 and lacks the ability to change intracapillary LPL levels or plasma triglyceride levels in *Apoa5*^*−/−*^ mice. Also, an antibody against the last 26 amino acids of APOA5 reduces intracapillary LPL levels and increases plasma triglyceride levels in wild-type mice. An inhibitory ANGPTL3/8-specific antibody functions as an APOA5-mimetic reagent, increasing intracapillary LPL levels and lowering plasma triglyceride levels in both *Apoa5*^*−/−*^ and wild-type mice. That antibody is a potentially attractive strategy for treating elevated plasma lipid levels in human patients.

In 2001, Pennacchio *et al.* (1) uncovered a new gene, *APOA5,* by comparative sequencing of the human and mouse *APOA1/APOC3/APOA4* gene cluster. Similarities between the structures of APOA5 and other apolipoproteins raised the possibility that APOA5 could play a role in lipoprotein metabolism. To explore that possibility, Pennacchio *et al.* (1) created *Apoa5-*deficient mice (*Apoa5*^−/−^) and transgenic mice that overexpressed human *APOA5* (*hAPOA5*-Tg). Plasma triglyceride (TG) levels were fourfold higher in *Apoa5*^−/−^ mice than in *Apoa5*^+/+^ mice and were 65% lower in *hAPOA5*-Tg mice than in the control mice (1). Subsequent studies revealed that loss-of-function mutations in human *APOA5* are associated with high plasma TG levels (2, 3, 4, 5, 6, 7) and an increased risk of atherosclerotic coronary artery disease (2, 8, 9).

The impressive effects of APOA5 on plasma TG levels prompted efforts to define the function of APOA5 in plasma lipid metabolism. Turnover studies involving radiolabeled TG-rich lipoproteins (TRLs) revealed that APOA5 promotes the clearance of TRLs (10, 11, 12, 13), but for years the underlying mechanism was unclear. One idea was that a positively charged heparin-binding domain in APOA5 interacts with heparan sulfate proteoglycans (HSPGs) on the luminal surface of capillary endothelial cells (ECs), thereby increasing the margination of APOA5-containing TRLs along capillaries and facilitating lipoprotein lipase (LPL)–mediated TG hydrolysis (12, 14). That proposal, however, was open to question because the plasma levels of APOA5 are extremely low (15, 16, 17, 18), such that few TRLs have even a single molecule of APOA5 (19, 20). Another idea was that APOA5 activates the catalytic activity of LPL (10, 11, 21), but several studies have been unable to detect any effect of APOA5 on LPL activity (12, 14, 22). Multiple groups have attempted to gauge the impact of APOA5 deficiency on LPL expression by measuring amounts of LPL activity in the plasma after a bolus of heparin. The results of those studies, both in humans and mice, have been inconclusive; several studies reported that post-heparin LPL levels were low (3, 4, 5, 13), whereas others concluded that the LPL levels were normal (6, 7, 12, 23).

Recent studies have clarified the function of APOA5. In vitro biochemical studies by Chen *et al.* (22) revealed that APOA5 binds to a physiologic inhibitor of LPL activity, the angiopoietin-like protein 3/8 complex (ANGPTL3/8) (24, 25, 26, 27, 28, 29), and suppresses ANGPTL3/8-mediated inhibition of LPL’s TG hydrolase activity. That observation implied that APOA5 deficiency would be accompanied by unsuppressed ANGPTL3/8 activity and increased inactivation of LPL.

A recent report by Yang *et al.* (30) added to our understanding of APOA5 and ANGPTL3/8 physiology. They found that amounts of LPL inside capillaries of the heart and brown adipose tissue (BAT) were lower in *Apoa5*^−/−^ mice than in *Apoa5*^+/+^ mice. That discovery implied that ANGPTL3/8 does more than simply inhibit the catalytic activity of LPL. Specifically, they proposed that ANGPTL3/8 functions to detach LPL from its binding sites within capillaries and that the detachment of LPL from capillaries is suppressed by APOA5 (30). Here, we review data that support that proposal. We will also discuss recent findings showing that carboxyl-terminal sequences in APOA5 are crucial for its ability to bind to ANGPTL3/8 and suppress ANGPTL3/8 activity (31). Finally, we discuss an inhibitory ANGPTL3/8-specific monoclonal antibody (mAb) that functions as an APOA5-mimetic reagent. In mice, the inhibitory mAb, like recombinant APOA5, increases intracapillary LPL levels and sharply reduces plasma TG levels (30). The inhibitory mAbs are likely to be therapeutically useful in humans. In a single-dose human trial, the inhibitory mAb sharply reduced plasma TG levels and led to substantial reductions in both LDL cholesterol (LDL-C), and APOB levels.

## APOA5 binds to ANGPTL3/8 and suppresses its capacity to inhibit LPL catalytic activity

ANGPTL3/8 is a physiologic inhibitor of LPL activity in oxidative tissues (25, 28, 32, 33). To screen for ANGPTL3/8-interacting proteins, Chen *et al.* (22) incubated human serum with ANGPTL3/8-coated beads, and the proteins that bound to the beads were digested with trypsin. Mass spectrometry–based studies revealed enrichment of APOA5 tryptic peptides (22). That finding raised the possibility that APOA5 binds to ANGPTL3/8 and that APOA5 could function to suppress ANGPTL3/8-mediated inhibition of LPL activity. To explore this idea, Chen *et al.* (22) produced recombinant human APOA5 and human ANGPTL3/8 (22); demonstrated that APOA5 binds to ANGPTL3/8 with high affinity (22); and showed that APOA5 suppresses the ability of ANGPTL3/8 to inhibit LPL activity (22). The binding of APOA5 to ANGPTL3/8 was specific. APOA5 did not bind or inhibit ANGPTL4 [which regulates LPL activity in adipose tissue (34, 35)], and published data suggest that APOA5 has little ability to suppress the capacity of ANGPTL3 [an inhibitor of LPL (36) and endothelial lipase (37)] to inhibit LPL catalytic activity (22).

In an independent study, Chen *et al.* (25) reported that ANGPTL3/8 binds to LPL, implying that ANGPTL3/8 could reduce LPL activity simply by binding to LPL and thereby interfering (in a direct fashion) with LPL’s ability to hydrolyze TRL triglycerides. Of note, APOA5 and LPL bind to similar sites on ANGPTL3/8 (38). With these observations in mind, one could propose that the binding of APOA5 to ANGPTL3/8 preserves LPL catalytic activity simply by interfering with ANGPTL3/8 binding to LPL. As it turned out, however, and as discussed later in this review, ANGPTL3/8’s impact on intravascular lipolysis is more complicated than simply binding to LPL and interfering with LPL’s ability to hydrolyze triglycerides. As explained later, ANGPTL3/8 reduces intracapillary LPL levels, and that effect is suppressed by APOA5.

## APOA5 deficiency results in reduced amounts of LPL within capillaries

**Fig. 1:**
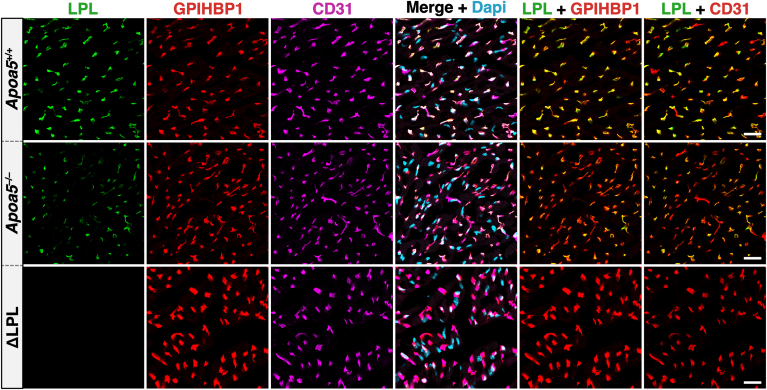
Confocal micrographs depicting amounts of LPL inside heart capillaries of *Apoa*5^+/+^ and *Apoa*5^−/−^ mice. To assess intracapillary LPL levels, *Apoa*5^−/−^ and *Apoa*5^+/+^ mice were given an intravenous injection of Alexa Fluor–labeled monoclonal antibodies (mAbs) against LPL, GPIHBP1, and CD31. After 10 min, cryosections of the heart were prepared for microscopy. Amounts of LPL on the luminal surface of capillaries, relative to amounts of GPIHBP1 and CD31, were assessed by quantifying fluorescent intensities. Fluorescence intensity ratio data are found in the paper by Yang *et al.* (30). Scale bars, 20 μm.

Recent studies by Yang *et al.* (30) added to our understanding of the mechanisms by which APOA5 and ANGPTL3/8 affect plasma TG metabolism. They found, by confocal microscopy, that amounts of LPL inside capillaries of oxidative tissues [*e.g.*, heart and brown adipose tissue (BAT)] are lower in *Apoa5*^−/−^ mice than in *Apoa5*^+/*+*^ mice (30). To quantify amounts of LPL on the luminal surface of endothelial cells (ECs), they gave *Apoa5*^−/−^ and *Apoa5*^+/*+*^ mice an intravenous injection of Alexa Fluor–labeled mAbs against LPL, GPIHBP1 (the LPL transporter in ECs), and CD31 (an EC protein) (39). Then, after 10 min, they perfused mice with PBS, perfusion-fixed the tissues, and prepared cryosections for fluorescence microscopy. The amounts of LPL, GPIHBP1, and CD31 in capillaries were assessed by quantifying Alexa Fluor signals in large numbers of capillary segments (n *=* 323–1870 segments/tissue/mouse in four independent experiments). These studies revealed that amounts of LPL inside capillaries, relative to GPIHBP1 or CD31, were significantly reduced in BAT and heart capillaries of *Apoa5*^−/−^ mice (Fig. 1). Consistent with these observations, they found reduced amounts of LPL mass and activity in the post-heparin plasma of *Apoa5*^−/−^ mice. Also, the margination of TRLs along the luminal surface of capillaries [a process that depends on intracapillary LPL (40, 41)] was lower in *Apoa5*^−/−^ mice than in *Apoa5*^+/*+*^ mice. These observations led Yang *et al.* (30) to propose that the in vivo effect of ANGPTL3/8 is more complex than simply binding to LPL and blocking LPL’s TG hydrolase activity. They proposed that ANGPTL3/8 detaches LPL from the luminal surface of capillaries and that the detachment of LPL is increased in the setting of APOA5 deficiency (where ANGPTL3/8 activity is unsuppressed).

The notion that ANGPTL3/8 could both inactivate LPL activity and detach LPL from capillaries was plausible. Earlier, Mysling *et al.* (42) proved, with hydrogen–deuterium exchange/mass spectrometry studies, that ANGPTL4 regulates LPL activity by *catalyzing* the unfolding of LPL’s hydrolase domain (resulting in irreversible loss of LPL catalytic activity). Follow-up studies by Leth-Espensen *et al.* (43) and Kumari *et al.* (44) demonstrated that ANGPTL4 binds to sequences surrounding LPL’s catalytic pocket and that this binding event initiates the unfolding of LPL’s native conformation, explaining the irreversible enzyme inactivation. Based on the ANGPTL4 precedent, Yang *et al.* (30) speculated that ANGPTL3/8 might unfold LPL conformation and that the ANGPTL3/8-mediated unfolding might account for both the loss of LPL catalytic activity and the detachment of LPL from the surface of capillaries. We emphasize, however, that this was merely speculation; the molecular mechanism underlying ANGPTL3/8 activity requires rigorous testing under a variety of experimental conditions.

## ANGPTL3/8 detaches LPL from the surface of cultured cells, and that detachment is blocked by APOA5 and by an inhibitory ANGPTL3/8-specific mAb

**Fig. 2:**
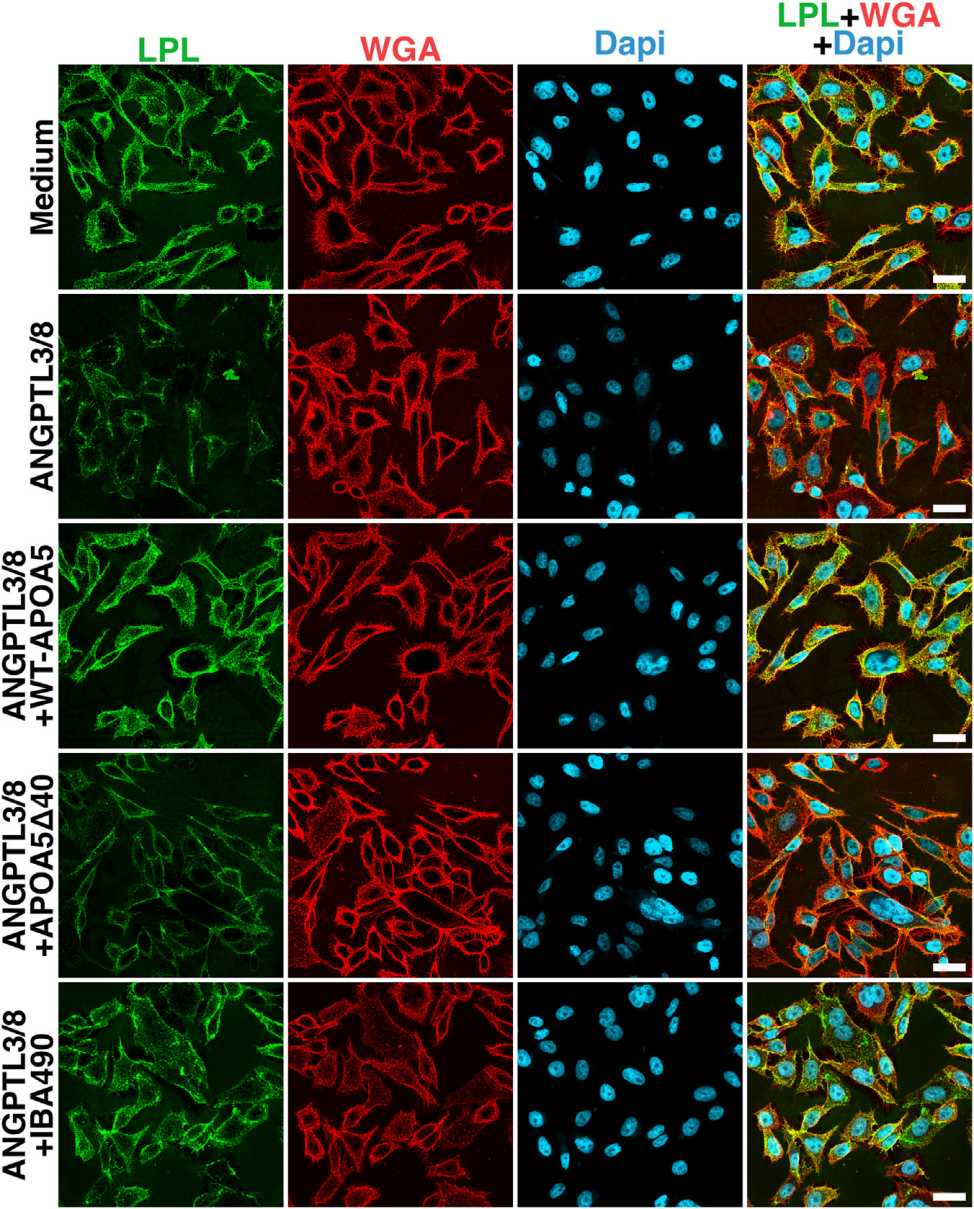
Confocal microscopy studies depicting the ability of recombinant ANGPTL3/8 to detach LPL from the surface of CHO-K1 cells and the capacities of WT-APOA5, APOA5Δ40 (a truncated mouse APOA5 lacking 40 C-terminal amino acids), and the inhibitory ANGPTL3/8-specific mAb IBA490 to suppress ANGPTL3/8-mediated LPL detachment. CHO-K1 cells were incubated with 50 nM human LPL for 10 min at 37°C. After washing, the cells were treated for 15 min at 37°C with serum-free cell culture medium alone; 100 nM ANGPTL3/8; 100 nM ANGPTL3/8 + 1.4 μM WT-mouse APOA5; 100 nM ANGPTL3/8 + 1.4 μM APOA5Δ40; or 100 nM ANGPTL3/8 + 1 μM IBA490. Amounts of LPL on the surface of nonpermeabilized cells were assessed by confocal microscopy after staining the cells with an Alexa Fluor 555–labeled mAb against LPL (5D2) and with Alexa Fluor 488–labeled wheat germ agglutinin (WGA). ANGPTL3/8 detached LPL, resulting in reduced amounts of human LPL on the surface of cells. WT-APOA5 and IBA490 suppressed ANGPTL3/8-mediated LPL detachment, whereas APOA5Δ40 did not. Quantification of the fluorescent dye intensities have been reported by Chen, Yang *et al.* (30, 31). Scale bars, 20 μm.

Yang *et al.* (45) tested whether recombinant ANGPTL3/8 was capable of detaching LPL from the surface of cultured cells, and if so, whether the detachment of LPL could be blocked by APOA5. First, they loaded cell-surface HSPGs of CHO-K1 cells with human LPL by incubating the cells with recombinant LPL. The cells were then washed and incubated with cell culture medium alone, ANGPTL3/8, or ANGPTL3/8 in the presence of either recombinant APOA5 or an inhibitory ANGPTL3/8-specific mAb (IBA490). IBA490 binds to an epitope in ANGPTL3/8 that overlaps with the APOA5 binding site (38). The surface of cells was then stained with an Alexa Fluor 555–labeled mAb against human LPL (5D2) and an Alexa Fluor 488–labeled wheat germ agglutinin (WGA, which binds to cell-surface HSPGs). Amounts of LPL on the surface of cells, as judged by 5D2 binding, were reduced in the cells that had been incubated with ANGPTL3/8 alone (Fig. 2). In cells that had been incubated with ANGPTL3/8 + APOA5 or ANGPTL3/8 + IBA490, amounts of LPL on the cell surface were not reduced. These findings provided a very plausible explanation for the reduced amounts of LPL along the luminal surface of capillaries in *Apoa5*^−/−^ mice.

## Inhibiting ANGPTL3/8 activity with either APOA5 or mAb IBA490 increases intracapillary LPL levels in *Apoa5*^−/−^ mice and normalizes plasma TG levels

**Fig. 3:**
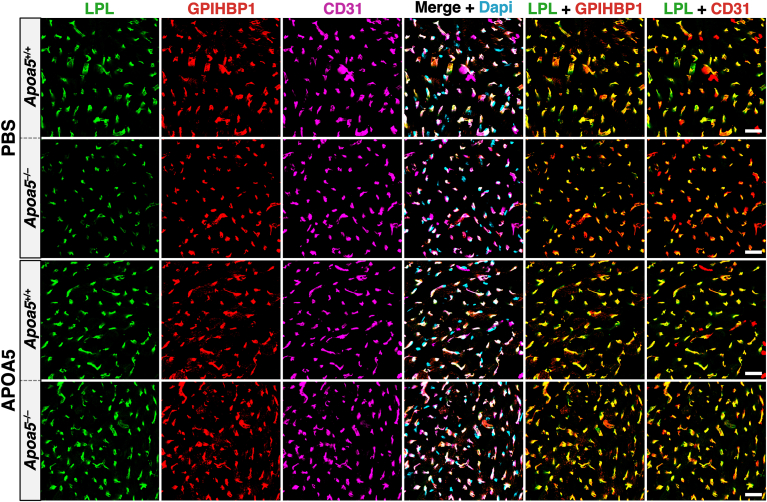
Confocal micrographs depicting amounts of LPL inside heart capillaries of *Apoa*5^−/−^ and *Apoa*5^+/+^ mice treated with recombinant APOA5 or phosphate-buffered saline (PBS) alone. *Apoa*5^−/−^ and *Apoa*5^+/+^ mice were given an intravenous injection of APOA5 (10 mg/kg) or PBS. Four h later, the amount of LPL inside heart capillaries in mice was measured by confocal microscopy, as described in Fig. 1. Intracapillary LPL levels in the heart were low in *Apoa5*^−/−^ mice and were increased by recombinant APOA5 (30). Scale bars, 20 μm.

**Fig. 4:**
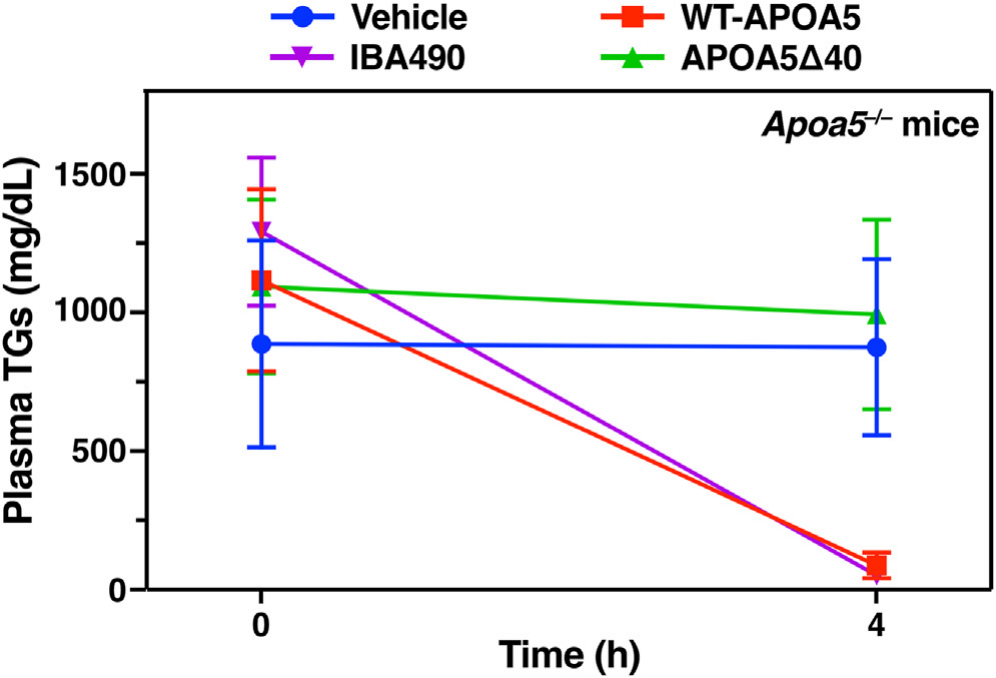
Testing the effects of recombinant mouse APOA5 proteins and an inhibitory ANGPTL3/8-specific antibody (IBA490) on plasma TG levels in *Apoa*5^−/−^ mice. *Apoa*5^−/−^ mice (n = 6–7/group) received an intravenous injection of 0.5 nmole of wild-type APOA5 (WT-APOA5), 0.5 nmole of APOA5Δ40 (a truncated APOA5 lacking 40 C-terminal residues), or vehicle (PBS) alone; two *Apoa*5^−/−^ mice were given an intravenous injection of the inhibitory ANGPTL3/8-specific mAb IBA490 (1.0 nmole). Plasma TG levels (mean ± SD) were determined at baseline and 4 h later. WT-APOA5 lowered plasma TG levels from 1116 ± 328 to 87 ± 47 mg/dl; IBA490 lowered TG levels from 1291 ± 268 to 52 ± 14 mg/dl. Plasma TG levels did not fall after treatment with APOA5Δ40 or PBS alone. This figure was generated from data reported by Chen, Yang, *et al.* (31).

The ability of APOA5 and IBA490 to suppress ANGPTL3/8-mediated detachment of LPL from cultured cells prompted Yang *et al.* (30) to test the impact of both APOA5 and IBA490 on intracapillary LPL levels in oxidative tissues in *Apoa5*^−/−^ mice. To explore the effects of APOA5, mice were given an intravenous injection of recombinant APOA5 or PBS alone. After 4 h, the mice were given an intravenous injection of Alexa Fluor–labeled mAbs against LPL, GPIHBP1, and CD31. After 10 min, cryosections of heart and BAT were prepared for confocal fluorescence microscopy. Amounts of LPL along the luminal surface of capillaries, relative to GPIHBP1 or CD31, were significantly increased in the *Apoa5*^−/−^ mice that had received recombinant APOA5 (30) (Fig. 3). Consistent with that finding, the plasma TG levels in the mice that had been given APOA5 fell dramatically (from 1116 to 87 mg/dl) (30) (Fig. 4).

**Fig. 5:**
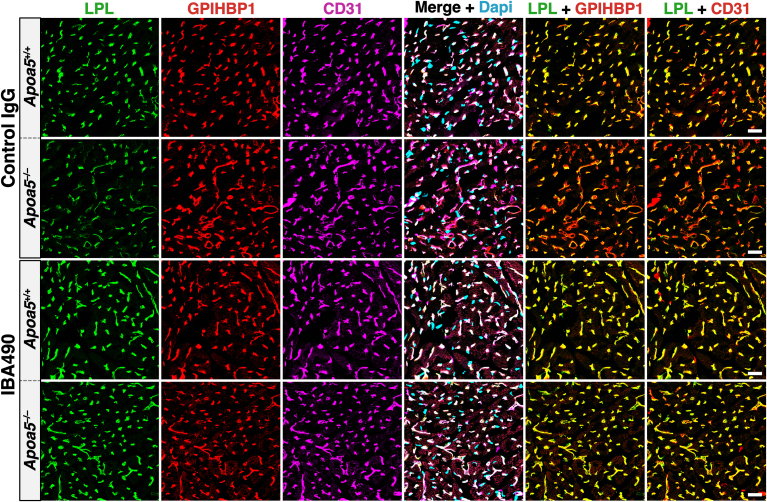
Confocal micrographs depicting amounts of LPL inside heart capillaries of *Apoa*5^+/+^ and *Apoa*5^−/−^ mice treated with either mAb IBA490 or an irrelevant control IgG. *Apoa*5^−/−^ and *Apoa*5^+/+^ mice were given a subcutaneous injection of IBA490 (10 mg/kg) or an irrelevant control IgG (10 mg/kg). After 24 h, amounts of intracapillary LPL in mice were assessed by fluorescent microscopy, as described in Fig. 1. Intracapillary LPL levels in the heart were low in *Apoa5*^−/−^ mice but were increased by mAb IBA490 (30). Scale bars, 20 μm.

To assess the impact of mAb IBA490 on TG metabolism, *Apoa5*^−/−^ mice were given a subcutaneous injection of IBA490 or an irrelevant control IgG (30). After 24 h, the mice were given an intravenous injection of Alexa Fluor–labeled mAbs against LPL, GPIHBP1, and CD31. After 10 min, the vasculature was perfused and fixed, and tissue cryosections were prepared for microscopy. Amounts of intracapillary LPL in the heart and BAT of *Apoa5*^−/−^ mice, relative to GPIHBP1 or CD31, were increased after IBA490 treatment (Fig. 5), and the plasma TG levels fell dramatically from 1291 to 52 mg/dl (30) (Fig. 4). The low plasma TG levels after IBA490 were sustained for three days (30).

In the microscopy studies designed to assess the impact of APOA5 and IBA490 on intracapillary LPL levels, the fluorescent signals for the mAbs against LPL, GPIHBP1, and CD31 were quantified in large numbers of capillaries (n = 381–2020 capillary segments/tissue/mouse in three independent experiments) (30). The increased intracapillary LPL levels after APOA5 and IBA490 were visibly apparent in the confocal micrographs and were confirmed by quantification of the fluorescent signals. For example, the LPL:GPIHBP1 and LPL:CD31 fluorescent intensity ratios in heart capillaries were significantly elevated in *Apoa5*^−/−^ mice that had been treated with APOA5 and IBA490 (30).

## Inhibiting ANGPTL3/8 activity with APOA5 or mAb IBA490 also increases intracapillary LPL levels in wild-type (*Apoa5*^+/+^) mice

The ability of APOA5 and IBA490 to increase intracapillary LPL levels was not confined to *Apoa5*^−/−^ mice. APOA5 and IBA490 infusions also increased intracapillary LPL levels in *Apoa5*^+/+^ mice, as judged by confocal microscopy–based measurements of LPL:GPIHBP1 fluorescent intensity ratios (30). The ability of IBA490 to influence intracapillary LPL levels in *Apoa5*^+/+^ mice was strongly supported by a study by Yang *et al.* (46) on levels of LPL inside heart capillaries during fasting and refeeding. More than three decades ago, Kuwajima *et al.* (47) demonstrated that LPL expression in the heart is high during fasting and low after refeeding, but the mechanism was unknown. We now know that refeeding triggers a ∼9-fold increase in the plasma levels of ANGPTL3/8 (25, 31). Yang *et al.* (46) showed, with immunofluorescence confocal microscopy, that amounts of LPL inside heart capillaries, relative to GPIHBP1 or CD31, are high in fasted *Apoa5*^+/+^ mice but fall by 50%–60% after refeeding. They also showed that the decrease in intracapillary LPL levels after refeeding can be abolished by inhibiting ANGPTL3/8 activity with mAb IBA490 (46).

We have not assessed the impact of APOA5 deficiency on the background of ANGPTL8 deficiency. Because APOA5 functions to suppress ANGPTL3/8 activity, our expectation is that APOA5 deficiency would have little impact on LPL levels in capillaries of heart and brown adipose tissue in *Angptl8*-deficient mice. However, interpreting such a model could be complicated by the absence of the ANGPTL4/8 complex in adipose tissue. An absence of the ANGPTL4/8 complex in adipose tissue could result in reduced amounts of intracapillary LPL in adipose tissue (29).

## Key Points

• APOA5 binds to the ANGPTL3/8 complex and suppresses its ability to inhibit LPL’s TG hydrolase activity.
• In *Apoa5*-deficient mice, unbridled ANGPTL3/8 activity results in reduced amounts of LPL along the luminal surface of heart and brown adipose tissue capillaries.
• ANGPTL3/8 detaches LPL from binding sites on the surface of cells; the detachment of LPL can be blocked by recombinant APOA5 and by an inhibitory antibody against ANGPTL3/8.
• APOA5’s ability to bind and suppress ANGPTL3/8 is utterly dependent on the last ∼40 amino acids of APOA5, explaining why *APOA5* truncation mutations in humans cause severe hypertriglyceridemia.
• An inhibitory ANGPTL3/8-specific monoclonal antibody mimics APOA5 function, preventing ANGPTL3/8-mediated inhibition of LPL activity and preserving amounts of LPL within capillaries.

## Carboxyl-terminal sequences in APOA5 are crucial for its ability to bind to ANGPTL3/8 and suppress ANGPTL3/8 activity

Clinical case reports described severe hypertriglyceridemia in two patients harboring an *APOA5* frameshift mutation that truncates APOA5 by 35 residues (48, 49). Those clinical findings inspired Chen, Yang, and coworkers to test whether the C-terminal sequences in human APOA5 are required for APOA5’s ability to suppress ANGPTL3/8 activity (31).

**Fig. 6:**
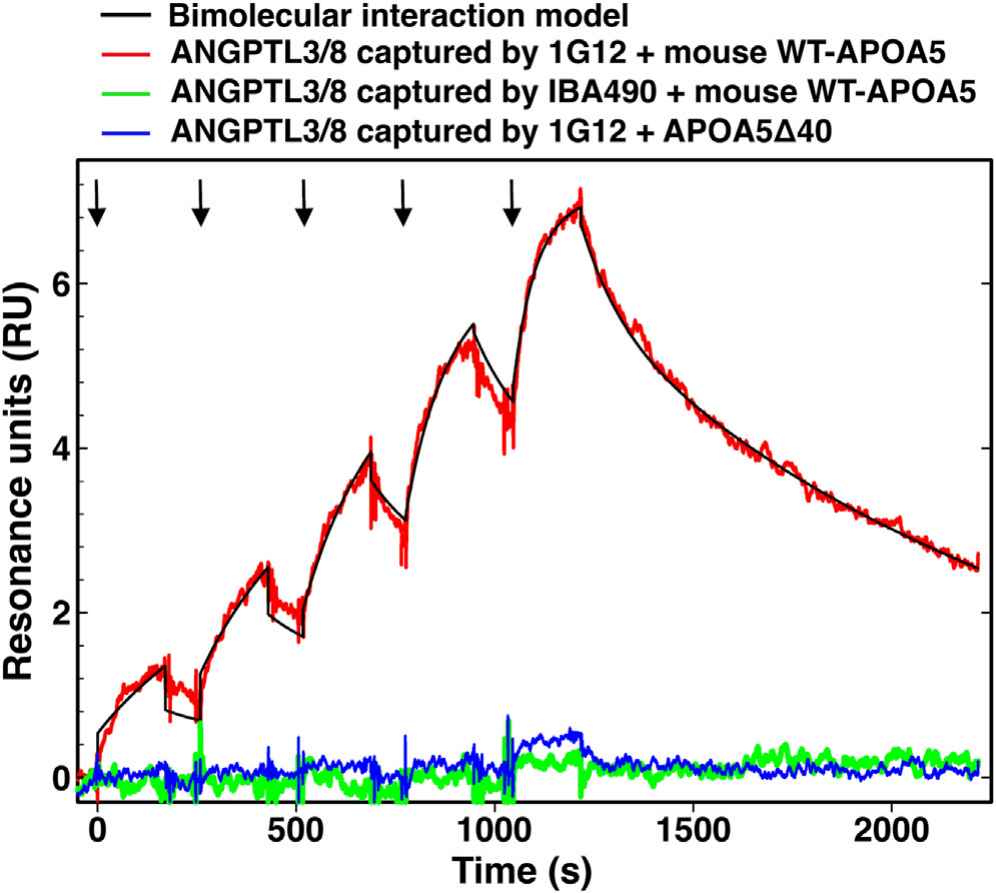
Testing the binding of mouse WT-APOA5 or APOA5Δ40 to ANGPTL3/8 by surface plasmon resonance (SPR). A CM4 sensor chip (coupled with a rabbit anti-mouse IgG) was primed with ANGPTL3/8-specific mAb 1G12 (which binds to the C-terminal fibrinogen-like domain) or mAb IBA490 (which binds to an epitope that overlaps with the APOA5 binding site). After an injection of 25 nM ANGPTL3/8, comparable amounts of ANGPTL3/8 were immobilized on the chip. The binding affinity of APOA5 proteins to immobilized ANGPTL3/8 was measured with injections of 1:2 serial dilutions of 0.5–8 nM WT-APOA5 or APOA5Δ40 (arrows). The binding of WT-APOA5 to 1G12-immobolized ANGPTL3/8 fits a simple bimolecular interaction model and yielded a dissociation constant (K_D_) of 0.53 nM, whereas the binding of APOA5Δ40 to 1G12-immobolized ANGPTL3/8 and the binding of WT-APOA5 to IBA490-captured ANGPTL3/8 were virtually undetectable. Reproduced with permission from Chen, Yang, *et al.* (31).

**Fig. 7:**
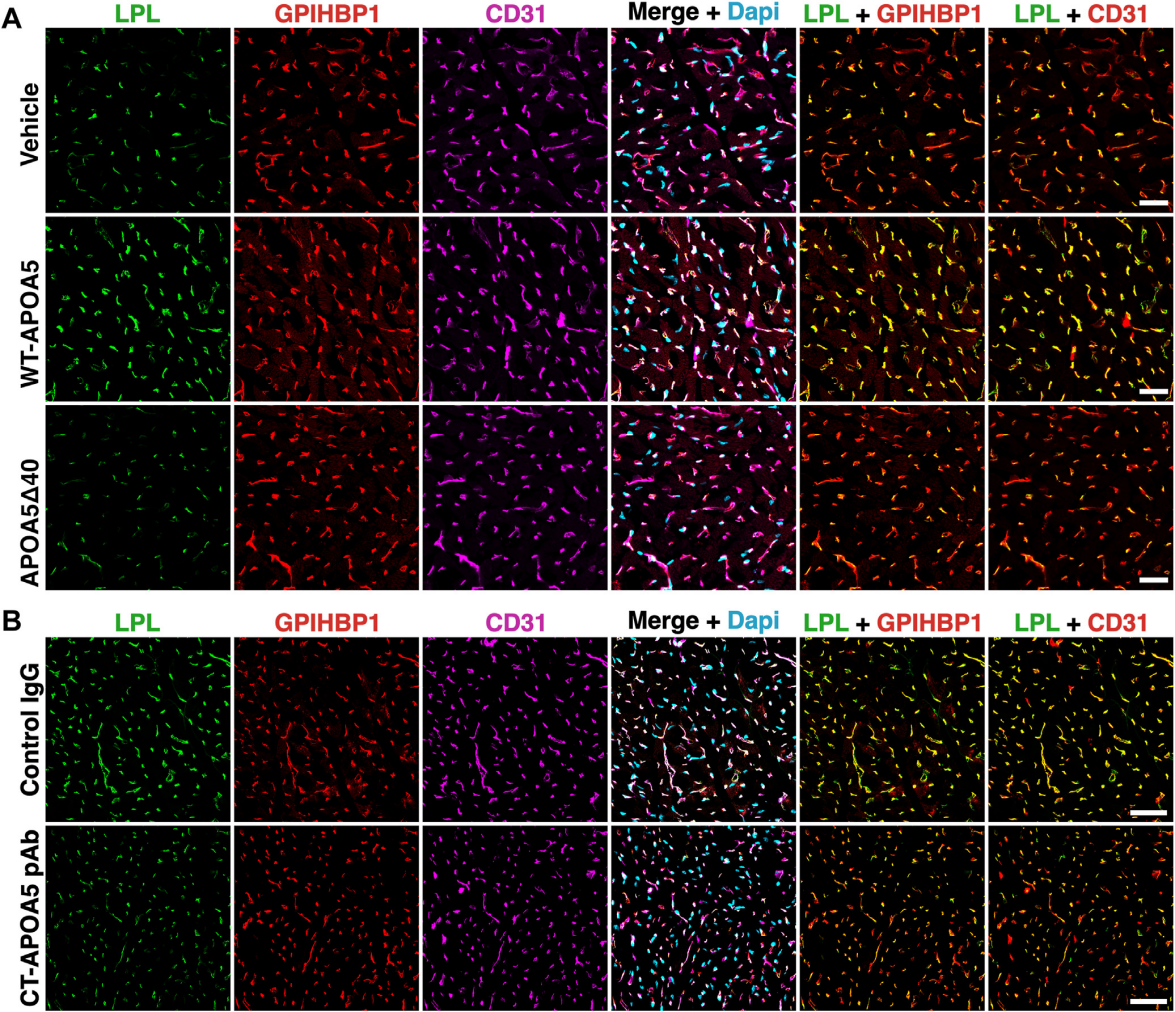
Confocal microscopy studies to assess the relevance of carboxyl-terminal APOA5 sequences for APOA5’s ability to regulate intracapillary LPL levels. A: *Apoa5*^−/−^ mice were given an injection of wild-type mouse APOA5 (WT-APOA5; 0.5 nmole/mouse), a truncated APOA5 lacking the last 40 residues of the protein (APOA5Δ40; 0.5 nmole/mouse), or vehicle (PBS) alone. After 4 h, the mice were given an intravenous injection of Alexa Fluor–labeled mAbs against LPL, GPIHBP1, and CD31. After 10 min, heart cryosections were prepared; and amounts of LPL, GPIHBP1, and CD31 on the luminal surface of capillaries were assessed by confocal microscopy and by quantifying LPL, GPIHBP1, and CD31 fluorescence intensities (31). WT-APOA5, but not APOA5Δ40, increased amounts of LPL in heart capillaries of *Apoa5*^−/−^ mice. Scale bars, 20 μm. B: fasted wild-type mice were given an intravenous injection of CT-APOA5 pAb (1.5 mg/mouse) or a nonimmune rabbit IgG (control IgG; 1.5 mg/mouse). After allowing the mice to refeed a chow diet for 6 h, the mice were given an intravenous injection of Alexa Fluor–labeled mAbs against LPL, GPIHBP1, and CD31. After 10 min, the tissues were fixed, and fluorescence intensities were imaged and quantified by confocal microscopy (31). The mice that had been treated with CT-APOA5 had reduced amounts of LPL in heart capillaries, consistent with the ability of CT-APOA5 pAb to neutralize the activity of APOA5. Scale bars, 50 μm. Reproduced with permission from Chen, Yang, *et al.* (31).

In vitro biochemical studies revealed that wild-type (WT) human APOA5, but not a mutant human APOA5 lacking 35 C-terminal amino acids (APOA5Δ35), blocked the ability of ANGPTL3/8 to inhibit LPL’s TG hydrolase activity. A truncated human APOA5 lacking 92 carboxyl-terminal residues also failed to suppress ANGPTL3/8 activity (31). To pursue these observations, Chen, Yang, *et al.* (31) introduced the “APOA5Δ35 frameshift mutation” into an expression vector for mouse APOA5 and then expressed and purified a truncated mouse APOA5 (APOA5Δ40). [The truncation mutation in mouse APOA5 results in the deletion of 40 residues rather than 35 (owing to a 5-residue extension at the C-terminus of mouse APOA5).] Surface plasmon resonance (SPR) studies revealed that WT mouse APOA5 bound to ANGPTL3/8 with high affinity (K_D_ = 0.53 nM), whereas there was no measurable binding of mouse APOA5Δ40 to ANGPTL3/8 (Fig. 6) (31). Consistent with that finding, ANGPTL3/8-mediated detachment of LPL from the surface of cultured cells was suppressed by WT mouse APOA5 (and by mAb IBA490) but not by APOA5Δ40 (Fig. 2). The cell culture experiments suggested that APOA5Δ40 would have no ability to influence plasma TG metabolism in *Apoa5*^−/−^ mice. Indeed, WT-APOA5, but not APOA5Δ40, sharply reduced plasma TG levels in *Apoa5*^−/−^ mice (Fig. 4). Also, WT-APOA5, but not APOA5Δ40, increased intracapillary LPL levels in the heart of *Apoa5*^−/−^ mice (Fig. 7A).

## An antibody against the last 26 residues of mouse APOA5 blocks the ability of APOA5 to suppress ANGPTL3/8 activity in vitro and in vivo

**Fig. 8:**
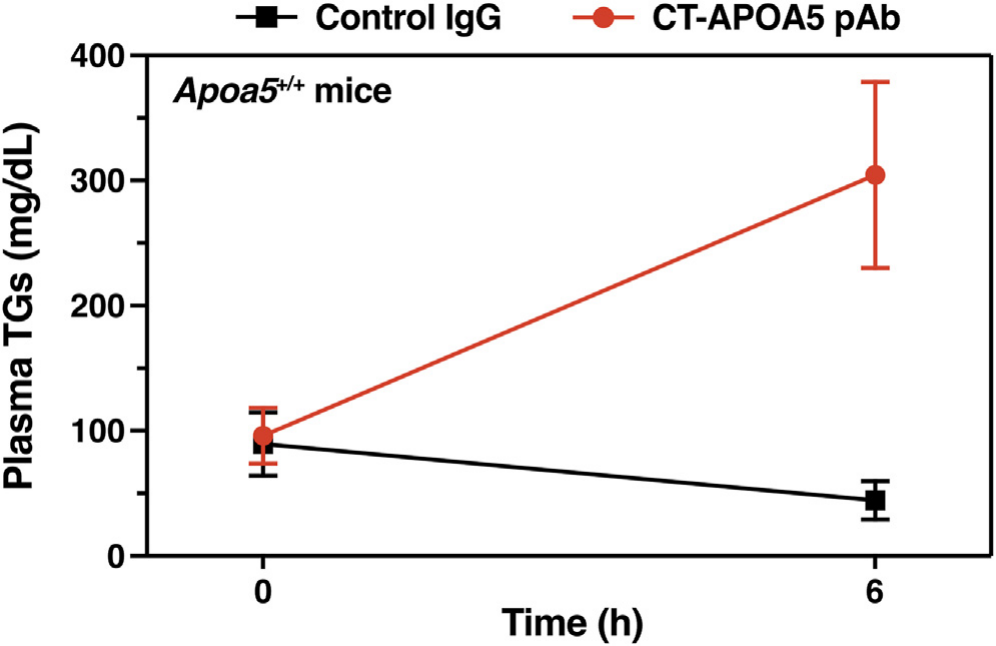
Testing the impact of a polyclonal antibody against the C-terminal 26 residues of APOA5 (CT-APOA5 pAb) on plasma TG levels in *Apoa*5^+/+^ mice. Fasted *Apoa*5^+/+^ mice (n = 7/group) were given an intravenous injection of CT-APOA5 pAb (1.5 mg/mouse) or a nonimmune rabbit IgG (control IgG; 1.5 mg/mouse) and then allowed to refeed a chow-diet. Plasma TG levels (mean ± SD) were measured at baseline and 6 h after the antibody injections. CT-APOA5 pAb increased plasma TG levels in *Apoa*5^+/+^ mice from 96 ± 22 mg/dl to 304 ± 74 mg/dl, whereas TG levels in mice that received control IgG remained low. Figures were created from data reported by Chen, Yang, *et al.* (31).

To further explore the idea that C-terminal sequences in APOA5 are crucial for regulating ANGPTL3/8 activity, Chen, Yang, and coworkers generated a synthetic peptide corresponding to the last 26 residues of mouse APOA5 and then made a rabbit polyclonal antibody, CT-APOA5 pAb, against the peptide (31). CT-APOA5 pAb bound to WT-mouse APOA5 but not to APOA5Δ40, as judged by western blot studies and immunohistochemistry studies on WT mouse liver (31). In vitro biochemical studies revealed that CT-APOA5 pAb abolished the ability of WT mouse APOA5 to suppress ANGPTL3/8-mediated inhibition of LPL catalytic activity (31). Also, CT-APOA5 pAb inhibited the ability of APOA5 to suppress ANGPTL3/8-mediated detachment of LPL from the surface of cultured cells (31). When CT-APOA5 pAb was infused into *Apoa5*^+/+^ mice, plasma TG levels increased from 96 to 304 mg/dl within 6 h, whereas an infusion of nonimmune rabbit IgG had no effect on plasma TG levels (Fig. 8) (31). Consistent with that observation, CT-APOA5 pAb, but not the control rabbit IgG, reduced amounts of LPL inside heart capillaries of *Apoa5*^+/+^ mice (Fig. 7B) (31).

The studies by Chen, Yang, and coworkers (31) revealed that C-terminal APOA5 sequences are crucial for the suppression of ANGPTL3/8 activity, but the identities of specific APOA5 amino acids that are important for the binding of APOA5 to ANGPTL3/8 have not yet been defined. Interestingly, AlphaFold3 predicts that the C-terminus of mouse APOA5 (S330–S358) contains an α-helix with hydrophobic residues (L334, L337, L341, L344, W345, I348, L352) on one surface of the helix. These residues are conserved in human and rat APOA5 (31, 50). While the confidence level for the AlphaFold3 structural predictions for APOA5’s C-terminal region was low, we nevertheless believe that it will be important, in future studies, to test whether the stretch of hydrophobic residues is crucial for APOA5’s ability to bind to ANGPTL3/8 and suppress its biological activity.

## IBA490 and APOA5 have the same effects on plasma TG metabolism

Recombinant APOA5 and IBA490 both suppress the ability of ANGPTL3/8 to inhibit LPL catalytic activity in vitro; both block ANGPTL3/8-mediated LPL detachment from cultured cells; both increase intracapillary LPL levels in heart and BAT; and both sharply reduce plasma TG levels in *Apoa5*^−/−^ mice (30). The fact that APOA5 and IBA490 have the same effects on intravascular lipolysis is not surprising. Earlier hydrogen–deuterium exchange/mass spectrometry studies revealed that the binding site on ANGPTL3/8 for a *human* ANGPTL3/8-specific mAb (5G11) overlaps with the binding site for APOA5 (38). [The variable domain of 5G11 and IBA490 are identical; IBA490 differs from 5G11 only by having a mouse Fc domain.] Because the binding sites for APOA5 and mAbs 5G11 and IBA490 on ANGPTL3/8 overlap and because APOA5 and the mAbs have the same effects on TG metabolism, 5G11 and IBA490 can be considered *APOA5-mimetic reagents.*

## Prospects for treating hyperlipidemic patients with an inhibitory ANGPTL3/8 mAb

The recent studies on APOA5 deficiency by Yang and coworkers (30) suggested that an inhibitory ANGPTL3/8 mAb (5G11) could be effective for treating patients with loss-of-function *APOA5* mutations. Effective therapies for APOA5 deficiency are important. Han Chinese populations from Southeast Asia harbor a pathogenic *APOA5* missense variant (p.G185C) that has been linked to elevated plasma TG levels (5) and an increased risk for coronary artery disease (9). That variant has an allele frequency of 7%, implying that there could be ∼100 million carriers in China alone.

The clinical utility of the inhibitory ANGPTL3/8 mAb could extend beyond patients with an inherited deficiency of APOA5. In wild-type mice, the mAb IBA490 resulted in higher intracapillary LPL levels and lower plasma TG levels (30). Also, increased expression of APOA5 (which functions to suppress ANGPTL3/8 activity) reduces plasma TG levels in wild-type mice (1, 10, 51).

The ability of mAb 5G11 to reduce plasma lipid levels was tested in a 28-day, randomized, double-blind, placebo-controlled trial of 48 human subjects with mixed hyperlipidemia (52). At the highest dose, 5G11 reduced plasma TG levels by 70%, remnant cholesterol levels by 61%, LDL-C levels by 36%, and APOB levels by 31% while increasing HDL cholesterol levels by 26% (52). Plasma TG lowering persisted for two weeks after the 5G11 infusion (52). Longer-term testing of 5G11 in different patient populations is needed, but it seems likely that mAb 5G11 will prove useful for treating a wide range of hyperlipidemia patients. The fact that 5G11 reduces plasma levels of both APOB and LDL-C raises the possibility that it could prove useful for reducing the risk of coronary heart disease.

## Conclusions

**Fig. 9:**
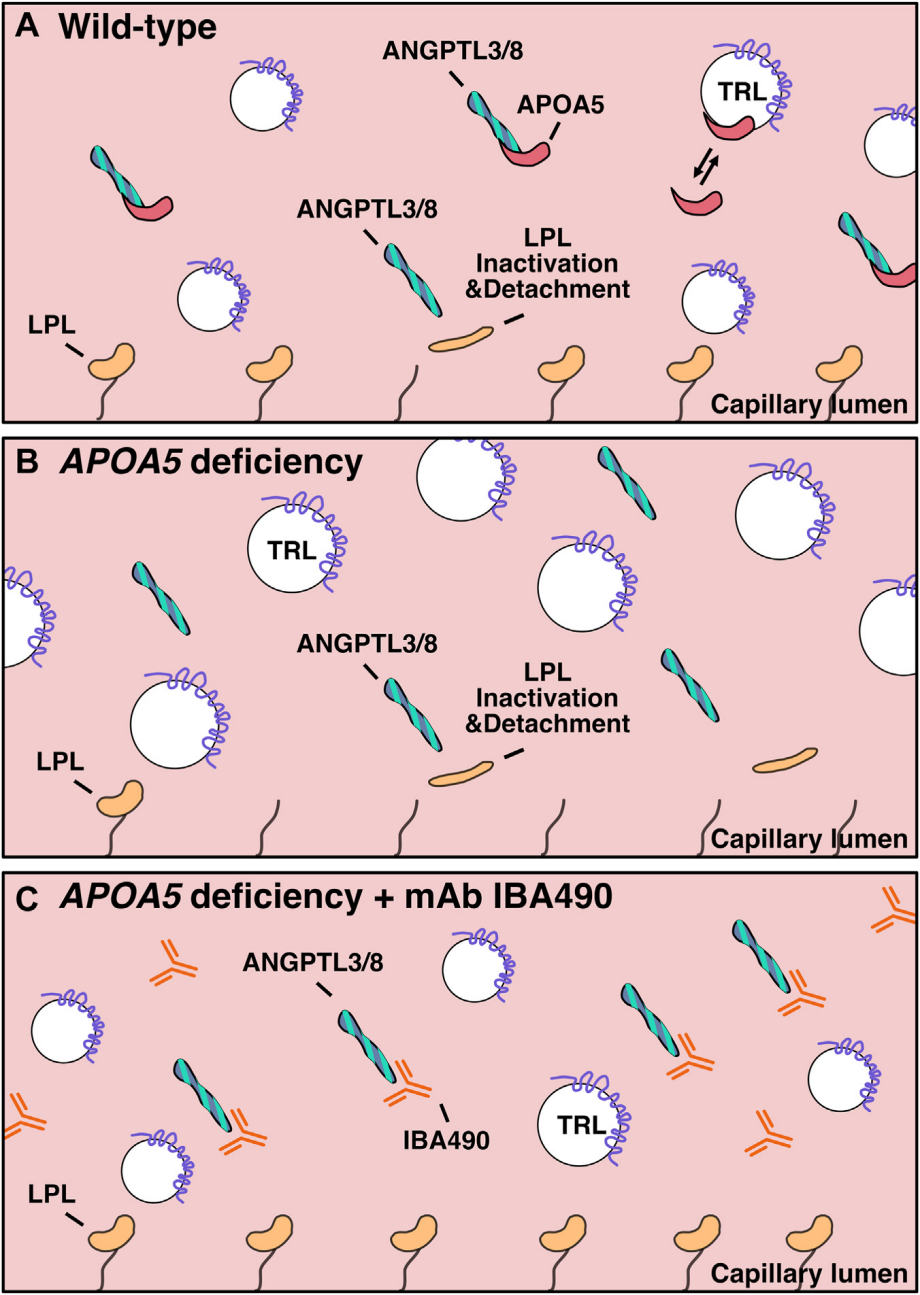
Schematic depictions of intracapillary triglyceride metabolism in wild-type mice, *Apoa5*^−/−^ mice, and *Apoa5*^−/−^ mice after an infusion of the inhibitory ANGPTL3/8-specific mAb IBA490. A: In wild-type mice, APOA5 binds to ANGPTL3/8 and suppresses its ability to inactivate LPL and blocks its ability to detach LPL from the luminal surface of capillary endothelial cells, thereby preserving robust lipolytic processing of triglyceride-rich lipoproteins (TRLs). B: In *Apoa5*^−/−^ mice, ANGPTL3/8 activity is increased because of the absence of APOA5. The increased ANGPTL3/8 activity inactivates LPL and detaches it from the surface of capillaries, thereby impeding the lipolytic processing of TRLs. C: In *Apoa5*^−/−^ mice, mAb IBA490 mimics the activity of APOA5. IBA490 binds to ANGPTL3/8 and suppresses ANGPTL3/8 activity, thereby increasing intracapillary LPL levels and augmenting the efficiency of TRL processing.

Now, two decades after the discovery of APOA5 and the discovery of severe hypertriglyceridemia in *Apoa5*^−/−^ mice (1), the molecular physiology of APOA5 has come into focus. APOA5 binds to ANGPTL3/8 and suppresses its ability to inhibit LPL-mediated processing of TRLs (Fig. 9A). In the setting of APOA5 deficiency, ANGPTL3/8 activity is unbridled, resulting in reduced inhibition of LPL catalytic activity in vitro and reduced amounts of intracapillary LPL in vivo (Fig. 9B). The inhibitory ANGPTL3/8-specific mAbs (IBA490, 5G11) are APOA5-mimetic agents; they suppress the ability of ANGPTL3/8 to inhibit LPL catalytic activity and to reduce intracapillary LPL levels (Fig. 9C). A study of 48 human subjects with mixed hyperlipidemia revealed that mAb 5G11 reduces plasma lipid levels of TGs, LDL-C, and APOB very effectively.

## Conflicts of interest

The authors declare the following financial interests/personal relationships which may be considered as potential competing interests: R. J. K. is an employee of Lilly Research Laboratories (Eli Lilly and Company) and holds stock and stock options in the company. S. G. Y. is on the scientific advisory board of Kyttaro and holds stock in that company (indicating a financial interest in that company).

## References

[1] Pennacchio L.A., Olivier M., Hubacek J.A., Cohen J.C., Cox D.R., Fruchart J.C. (2001). An apolipoprotein influencing triglycerides in humans and mice revealed by comparative sequencing. Science.

[2] Hubacek J.A., Skodová Z., Adámková V., Lánská V., Poledne R. (2004). The influence of APOAV polymorphisms (T-1131>C and S19>W) on plasma triglyceride levels and risk of myocardial infarction. Clin. Genet..

[3] Marçais C., Verges B., Charrière S., Pruneta V., Merlin M., Billon S. (2005). APOA5 Q139X truncation predisposes to late-onset hyperchylomicronemia due to lipoprotein lipase impairment. J. Clin. Invest..

[4] Priore Oliva C., Pisciotta L., Li Volti G., Sambataro M.P., Cantafora A., Bellocchio A. (2005). Inherited apolipoprotein AV deficiency in severe hypertriglyceridemia. Arterioscler. Thromb. Vasc. Biol..

[5] Pullinger C.R., Aouizerat B.E., Movsesyan I., Durlach V., Sijbrands E.J., Nakajima K. (2008). An apolipoprotein AV gene SNP is associated with marked hypertriglyceridemia among Asian-American patients. J. Lipid Res..

[6] Priore Oliva C., Carubbi F., Schaap F.G., Bertolini S., Calandra S. (2008). Hypertriglyceridemia and low plasma HDL in a patient with apolipoprotein AV deficiency due to a novel mutation in the APOA5 gene. J. Intern. Med..

[7] Albers K., Schlein C., Wenner K., Lohse P., Bartelt A., Heeren J. (2014). Homozygosity for a partial deletion of apolipoprotein AV signal peptide results in intracellular missorting of the protein and chylomicronemia in a breast-fed infant. Atherosclerosis.

[8] Do R., Stitziel N.O., Won H.H., Jørgensen A.B., Duga S., Angelica Merlini P. (2015). Exome sequencing identifies rare LDLR and APOA5 alleles conferring risk for myocardial infarction. Nature.

[9] Han Y., Dorajoo R., Chang X., Wang L., Khor C.C., Sim X. (2017). Genome-wide association study identifies a missense variant at APOA5 for coronary artery disease in multi-ethnic cohorts from southeast Asia. Sci. Rep..

[10] Schaap F.G., Rensen P.C., Voshol P.J., Vrins C., van der Vliet H.N., Chamuleau R.A. (2004). APOAV reduces plasma triglycerides by inhibiting very low density lipoprotein-triglyceride (VLDL-TG) production and stimulating lipoprotein lipase-mediated VLDL-TG hydrolysis. J. Biol. Chem..

[11] Fruchart-Najib J., Baugé E., Niculescu L.S., Pham T., Thomas B., Rommens C. (2004). Mechanism of triglyceride lowering in mice expressing human apolipoprotein A5. Biochem. Biophys. Res. Commun..

[12] Merkel M., Loeffler B., Kluger M., Fabig N., Geppert G., Pennacchio L.A. (2005). Apolipoprotein AV acelerates plasma hydrolysis of triglyceride-rich lipoproteins by interaction with proteoglycan-bound lipoprotein lipase. J. Biol. Chem..

[13] Grosskopf I., Baroukh N., Lee S.J., Kamari Y., Harats D., Rubin E.M. (2005). Apolipoprotein AV deficiency results in marked hypertriglyceridemia attributable to decreased lipolysis of triglyceride-rich lipoproteins and removal of their remnants. Arterioscler. Thromb. Vasc. Biol..

[14] Lookene A., Beckstead J.A., Nilsson S., Olivecrona G., Ryan R.O. (2005). Apolipoprotein AV–heparin interactions: implications for plasma lipoprotein metabolism. J. Biol. Chem..

[15] van der Vliet H.N., Sammels M.G., Leegwater A.C., Levels J.H., Reitsma P.H., Boers W. (2001). Apolipoprotein AV: a novel apolipoprotein associated with an early phase of liver regeneration. J. Biol. Chem..

[16] O'Brien P.J., Alborn W.E., Sloan J.H., Ulmer M., Boodhoo A., Knierman M.D. (2005). The novel apolipoprotein A5 is present in human serum, is associated with VLDL, HDL, and chylomicrons, and circulates at very low concentrations compared with other apolipoproteins. Clin. Chem..

[17] Ishihara M., Kujiraoka T., Iwasaki T., Nagano M., Takano M., Ishii J. (2005). A sandwich enzyme-linked immunosorbent assay for human plasma apolipoprotein AV concentration. J. Lipid Res..

[18] Alborn W.E., Johnson M.G., Prince M.J., Konrad R.J. (2006). Definitive N-terminal protein sequence and further characterization of the novel apolipoprotein A5 in human serum. Clin. Chem..

[19] Merkel M., Heeren J. (2005). Give me A5 for lipoprotein hydrolysis. J. Clin. Invest..

[20] Olofsson S.O. (2005). APOAV: the regulation of a regulator of plasma triglycerides. Arterioscler. Thromb. Vasc. Biol..

[21] Sun G., Bi N., Li G., Zhu X., Zeng W., Wu G. (2006). Identification of lipid binding and lipoprotein lipase activation domains of human APOAV. Chem. Phys. Lipids.

[22] Chen Y.Q., Pottanat T.G., Zhen E.Y., Siegel R.W., Ehsani M., Qian Y.W. (2021). APOA5 lowers triglyceride levels via suppression of ANGPTL3/8-mediated LPL inhibition. J. Lipid Res..

[23] Takanashi M., Kimura T., Li C., Tanaka M., Matsuhashi A., Yoshida H. (2019). Critical role of SREBP-1c large-VLDL pathway in environment-induced hypertriglyceridemia of APOAV deficiency. Arterioscler. Thromb. Vasc. Biol..

[24] Wang Y., Quagliarini F., Gusarova V., Gromada J., Valenzuela D.M., Cohen J.C. (2013). Mice lacking ANGPTL8 (Betatrophin) manifest disrupted triglyceride metabolism without impaired glucose homeostasis. Proc. Natl. Acad. Sci. U. S. A..

[25] Chen Y.Q., Pottanat T.G., Siegel R.W., Ehsani M., Qian Y.W., Zhen E.Y. (2020). Angiopoietin-like protein 8 differentially regulates ANGPTL3 and ANGPTL4 during postprandial partitioning of fatty acids. J. Lipid Res..

[26] Wen Y., Chen Y.Q., Konrad R.J. (2024). Angiopoietin-like protein 8: a multifaceted protein instrumental in regulating triglyceride metabolism. Curr. Opin. Lipidol..

[27] Wang Y., McNutt M.C., Banfi S., Levin M.G., Holland W.L., Gusarova V. (2015). Hepatic ANGPTL3 regulates adipose tissue energy homeostasis. Proc. Natl. Acad. Sci. U. S. A..

[28] Haller J.F., Mintah I.J., Shihanian L.M., Stevis P., Buckler D., Alexa-Braun C.A. (2017). ANGPTL8 requires ANGPTL3 to inhibit lipoprotein lipase and plasma triglyceride clearance. J. Lipid Res..

[29] Oldoni F., Cheng H., Banfi S., Gusarova V., Cohen J.C., Hobbs H.H. (2020). ANGPTL8 has both endocrine and autocrine effects on substrate utilization. JCI Insight.

[30] Yang Y., Beigneux A.P., Song W., Nguyen L.P., Jung H., Tu Y. (2023). Hypertriglyceridemia in *Apoa5*^–/–^ mice results from reduced amounts of lipoprotein lipase in the capillary lumen. J. Clin. Invest..

[31] Chen Y.Q., Yang Y., Zhen E.Y., Beyer T.P., Li H., Wen Y. (2024). Carboxyl-terminal sequences in APOA5 are important for suppressing ANGPTL3/8 activity. Proc. Natl. Acad. Sci. U. S. A..

[32] Quagliarini F., Wang Y., Kozlitina J., Grishin N.V., Hyde R., Boerwinkle E. (2012). Atypical angiopoietin-like protein that regulates ANGPTL3. Proc. Natl. Acad. Sci. U. S. A..

[33] Chi X., Britt E.C., Shows H.W., Hjelmaas A.J., Shetty S.K., Cushing E.M. (2017). ANGPTL8 promotes the ability of ANGPTL3 to bind and inhibit lipoprotein lipase. Mol. Metab..

[34] Kroupa O., Vorrsjö E., Stienstra R., Mattijssen F., Nilsson S.K., Sukonina V. (2012). Linking nutritional regulation of ANGPTL4, GPIHBP1, and LMF1 to lipoprotein lipase activity in rodent adipose tissue. BMC Physiol..

[35] Cushing E.M., Chi X., Sylvers K.L., Shetty S.K., Potthoff M.J., Davies B.S.J. (2017). Angiopoietin-like 4 directs uptake of dietary fat away from adipose during fasting. Mol. Metab..

[36] Shimizugawa T., Ono M., Shimamura M., Yoshida K., Ando Y., Koishi R. (2002). ANGPTL3 decreases very low density lipoprotein triglyceride clearance by inhibition of lipoprotein lipase. J. Biol. Chem..

[37] Shimamura M., Matsuda M., Yasumo H., Okazaki M., Fujimoto K., Kono K. (2007). Angiopoietin-like protein3 regulates plasma HDL cholesterol through suppression of endothelial lipase. Arterioscler. Thromb. Vasc. Biol..

[38] Balasubramaniam D., Schroeder O., Russell A.M., Fitchett J.R., Austin A.K., Beyer T.P. (2022). An anti-ANGPTL3/8 antibody decreases circulating triglycerides by binding to a LPL-inhibitory leucine zipper-like motif. J. Lipid Res..

[39] Song W., Yang Y., Heizer P., Tu Y., Weston T.A., Kim J.R. (2023). Intracapillary LPL levels in brown adipose tissue, visualized with an antibody-based approach, are regulated by ANGPTL4 at thermoneutral temperatures. Proc. Natl. Acad. Sci. U. S. A..

[40] Goulbourne C.N., Gin P., Tatar A., Nobumori C., Hoenger A., Jiang H. (2014). The GPIHBP1-LPL complex is responsible for the margination of triglyceride-rich lipoproteins in capillaries. Cell Metab..

[41] Song W., Beigneux A.P., Weston T.A., Chen K., Yang Y., Nguyen L.P. (2023). The lipoprotein lipase that is shuttled into capillaries by GPIHBP1 enters the glycocalyx where it mediates lipoprotein processing. Proc. Natl. Acad. Sci. U. S. A..

[42] Mysling S., Kristensen K.K., Larsson M., Kovrov O., Bensadouen A., Jørgensen T.J. (2016). The angiopoietin-like protein ANGPTL4 catalyzes unfolding of the hydrolase domain in lipoprotein lipase and the endothelial membrane protein GPIHBP1 counteracts this unfolding. Elife.

[43] Leth-Espensen K.Z., Kristensen K.K., Kumari A., Winther A.L., Young S.G., Jorgensen T.J.D. (2021). The intrinsic instability of the hydrolase domain of lipoprotein lipase facilitates its inactivation by ANGPTL4-catalyzed unfolding. Proc. Natl. Acad. Sci. U. S. A..

[44] Kumari A., Gronnemose A.L., Kristensen K.K., Winther A.L., Young S.G., Jorgensen T.J.D. (2023). Inverse effects of APOC2 and ANGPTL4 on the conformational dynamics of lid-anchoring structures in lipoprotein lipase. Proc. Natl. Acad. Sci. U. S. A..

[45] Berryman D.E., Bensadoun A. (1995). Heparan sulfate proteoglycans are primarily responsible for the maintenance of enzyme activity, binding, and degradation of lipoprotein lipase in Chinese hamster ovary cells. J. Biol. Chem..

[46] Yang Y., Jung H., Konrad R.J., Fong L.G., Young S.G. (2023). Imaging the ANGPTL3/8-mediated regulation of lipoprotein lipase in the heart. J. Lipid Res..

[47] Kuwajima M., Foster D.W., McGarry J.D. (1988). Regulation of lipoprotein lipase in different rat tissues. Metabolism.

[48] Mendoza-Barberá E., Julve J., Nilsson S.K., Lookene A., Martín-Campos J.M., Roig R. (2013). Structural and functional analysis of APOA5 mutations identified in patients with severe hypertriglyceridemia. J. Lipid Res..

[49] Hooper A.J., Kurtkoti J., Hamilton-Craig I., Burnett J.R. (2014). Clinical features and genetic analysis of three patients with severe hypertriglyceridaemia. Ann. Clin. Biochem..

[50] Abramson J., Adler J., Dunger J., Evans R., Green T., Pritzel A. (2024). Accurate structure prediction of biomolecular interactions with AlphaFold 3. Nature.

[51] van der Vliet H.N., Schaap F.G., Levels J.H., Ottenhoff R., Looije N., Wesseling J.G. (2002). Adenoviral overexpression of apolipoprotein AV reduces serum levels of triglycerides and cholesterol in mice. Biochem. Biophys. Res. Commun..

[52] Gaudet D., Gonciarz M., Shen X., Mullins G., Leohr J., Benichou O. (2022). A first-in-human single ascending dose study of a monoclonal antibody against the ANGPLT3/8 complex in subjects with mixed hyperlipidemia. Atherosclerosis.

